# Profound Hypoglycemia and High Anion Gap Metabolic Acidosis in a Pediatric Leukemic Patient Receiving 6-Mercaptopurine

**DOI:** 10.3390/children11020160

**Published:** 2024-01-26

**Authors:** Molly O’Shea, Alexis Kuhn, Ana L. Creo, Mira Kohorst, Asmaa Ferdjallah

**Affiliations:** 1 Mayo Clinic Alix School of Medicine, Rochester, MN 55905, USA; oshea.mary@mayo.edu; 2 Pediatric Hematology and Oncology, Mayo Clinic, Rochester, MN 55905, USA; kuhn.alexis@mayo.edu (A.K.); kohorst.mira@mayo.edu (M.K.); 3 Pediatric Endocrinology, Mayo Clinic, Rochester, MN 55905, USA; creo.ana@mayo.edu

**Keywords:** acute lymphoblastic leukemia, hypoglycemia, 6-mercaptopurine, anion gap metabolic acidosis

## Abstract

A 13-year-old male undergoing maintenance chemotherapy with methotrexate and 6-mercaptopurine (6MP), for very high-risk B-cell acute lymphoblastic leukemia (ALL), presented with vomiting due to severe hypoglycemia with metabolic acidosis. While his laboratory values were concerning for a critically ill child, the patient was relatively well appearing. Hypoglycemia is a rare but serious side effect of 6MP with an unexpectedly variable presentation; therefore, a high index of suspicion is needed for its prompt detection and treatment. This patient also had severe metabolic acidosis, likely secondary to hypoglycemia, creating a serious clinical picture despite a well-appearing child. This example of incongruity between laboratory tests and clinical appearance adds nuance to the existing literature. Moreover, although 6MP-associated hypoglycemia is rare, it may be more prevalent than the literature suggests, as symptoms of hypoglycemia—nausea, vomiting, and somnolence—mirror common chemotherapy side effects. 6MP-induced hypoglycemia can be ameliorated with the addition of allopurinol to shunt metabolism in favor of the production of therapeutic metabolites over hepatotoxic metabolites. Additionally, a morning administration of 6MP and frequent snacks may also help to prevent hypoglycemia. Overall, this case adds to the literature of unusual reactions to 6MP including hypoglycemia in an older child without traditional risk factors.

## 1. Introduction

6-mercaptopurine (6MP) is an integral drug in the treatment of acute lymphoblastic leukemia (ALL) and, along with methotrexate, steroids, and vincristine, forms the backbone of maintenance therapy [1]. Treatment of ALL typically consists of several phases including induction, consolidation, and maintenance, which include 6MP at various time points [2,3]. 6MP is used most regularly during the maintenance phase of therapy and is a purine analog with anti-neoplastic and immunosuppressive properties [2,3]. Its use has accounted for the excellent overall survival of pediatric patients with ALL of over 90%. Common side effects of 6MP include myelosuppression and hepatotoxicity, as well as fatigue, nausea, vomiting, rash, and decreased appetite [4]. More rarely, 6MP can induce severe hypoglycemia. Hepatotoxicity can present as a transient or asymptomatic rise in serum aminotransferase, alkaline phosphatase level, or total bilirubin, leading to significant jaundice [5]. 

The presentation of hypoglycemia is variable, including signs and symptoms such as anxiety, confusion, light-headedness, weakness, tremor, diaphoresis, and tachycardia [4,6]. These nonspecific symptoms of a dangerous condition therefore require high clinical suspicion to initiate appropriate treatment [6]. These findings are particularly challenging as they may lead to non-adherence and dose reduction in otherwise necessary chemotherapy [1].

We describe the case of a 13-year-old male with high-risk B-cell ALL who developed sudden and severe hypoglycemia while undergoing maintenance chemotherapy with 6MP. His laboratory values revealed severe metabolic acidosis and severe hypoglycemia, yet he presented as a well-appearing child. He subsequently required dose modification to tolerate 6MP and is currently doing well and remains in remission. The incongruity of his laboratory values and his clinical presentation highlight the importance of maintaining a high index of suspicion for hypoglycemia in patients receiving 6MP to ensure prompt treatment in the absence of alarming symptoms.

## 2. Case Description

A 13-year-old male undergoing cycle 5 of maintenance chemotherapy with methotrexate, 6MP, vincristine, and prednisone as per the Children’s Oncology Group protocol AALL1131 for very high-risk B-cell ALL presented to his local Emergency Department (ED) with non-bloody and non-bilious vomiting and a glucose of 47 mg/dL on home glucose testing (nl 70–100 mg/dL) [7,8]. Despite having received 6MP at multiple time points during his leukemia therapy, he had not had episodes of hypoglycemia in the past. In the ED, he was found to be tachycardic with a point-of-care glucose of 30 mg/dL. A physical exam revealed a well-appearing child in no acute distress. Significant findings included dry mucous membranes and right lower quadrant tenderness following an uncomplicated appendectomy 2 months prior. Two peripheral intravenous (IV) catheters were placed, and he received 25 g of D50 W and two 1 L normal saline boluses. He was empirically administered 1 mg/kg stress dose of hydrocortisone and 1 g dose of ceftriaxone.

Further labs revealed a low bicarbonate of 10 mmol/L (nl 22–27), anion gap 33 (nl 7–15), aspartate aminotransferase (AST) 197 IU/L (nl 10–40 IU/L), alanine aminotransferase (ALT) 256 IU/L (nl 10–40 IU/L), total bilirubin 1.1 mg/dL (nl < 1.0 mg/dL), and creatinine 0.41 mg/dL (nl 0.74–1.35 mg/dL) (see Table 1). A venous blood gas revealed a pH of 7.07 (nl 7.32–7.43), CO_2_ of 33 mmHg (40–60 mmHg), and a bicarbonate of 9 mmol/L (nl 22–27 mmol/L). An elevated lactate of 8.7 mmol/L (nl < 2.0 mmol/L) raised concern for a smoldering intrabdominal abscess, particularly given a recent history of appendectomy. An abdominal CT was unremarkable and did not identify an abscess. A beta hydroxybutyrate was obtained of 2.8 mmol/L (nl 0–0.5 mmol/L), and a urinalysis revealed elevated ketones (>150 mg/dL); both of which were consistent with his known severe hypoglycemia. His cortisol was 36 mcg/dL (nl 2.5–12 mcg/dL). In addition to the metabolic derangements, he was found to be neutropenic with an ANC of 320 × 10^9^/L (nl 2500–6000 × 10^9^/L) and thrombocytopenic with platelets of 50 × 10^9^/L (nl 150–450 × 10^9^/L). Thiopurine metabolites were also evaluated at the time, revealing mildly elevated 6-Thioguanine Nucleotide (6TGNs) of 585 p/mol/8 × 10^8^ RBC (nl 235–450 p/mol/8 × 10^8^ RBC) and markedly elevated 6-Methylmercaptopurine nucleotides (6MMPNs) of >52,320 pmol/8 × 10^8^ RBC (nl ≤ 5700 pmol/8 × 10^8^ RBC).

He was determined to have evidence of a severe high anion gap metabolic acidosis in the setting of hypoglycemia. The differential diagnosis remained broad, and, most likely, etiologies included medication side effects and infection. Given his recent appendectomy and accompanying abdominal tenderness, a smoldering abdominal abscess was of concern as it could cause nausea, vomiting, abdominal tenderness, and elevated lactate. His abdominal exam was non-focal and non-peritonitic. His normal temperature and the absence of an acute abdomen made an infectious etiology less likely. Additional possible causes of such metabolic derangements included regional ischemia, global ischemia, diabetic ketoacidosis, toxins, and high metabolic states (i.e., seizure, heavy exercise). Given the patient’s history of administering 6MP at night, low glucose level, elevated lactate level, and profoundly elevated 6MMPNs, the clinical picture as a whole suggested medication toxicity as the leading differential. He was admitted to the inpatient hospital service for observation, and his oral chemotherapies were temporarily discontinued. Interestingly, his acidosis corrected with only 2 L boluses of normal saline and holding 6MP. These interventions alone would otherwise not have been expected to have resolved his severe blood gas, bicarbonate, and lactate.

The patient’s thiopurine methyltransferase (TPMT) activity profile at diagnosis revealed him to be a normal metabolizer of 6MP. The TPMT profile can be either normal, intermediate, or poor, and normal metabolizers have an increased risk of 6MMP hepatotoxicity [9]. The patient’s methylmercaptopurine (6MMP)/6-thioguanine nucleotide (6TGN) ratio was >90. A ratio of >20 is associated with increased risk of hepatotoxicity [9]. In response to his aberrant 6MP metabolic profile, his 6MP dose was reduced, and allopurinol was initiated to shunt 6MP metabolism and increase thioguanine production relative to 6MMP production. Once his ANC recovered 21 days after his initial presentation, 6MP was resumed at 255 mg/wk (159 mg/m^2^). This was a dose reduction of 30% of the previous dose with the addition of allopurinol 100 mg (62 mg/m^2^) daily. While dose escalating the 6MP dose, he became neutropenic once again, prompting another hold of 6MP and allopurinol. Allopurinol was then re-initiated at 50 mg daily (31 mg/m^2^) with an additional 50% reduction of the already reduced 6MP dose to 127.5 mg/wk (79 mg/m^2^). Following the addition of allopurinol and further dose reduction of 6MP, he did not experience another hypoglycemic episode. Subsequent thiopurine metabolites identified a markedly improved metabolic profile with 6TGNs of 371 pmol/8 × 10^8^ RBC and 6MMPNs below the limit of detection in the setting of an appropriate degree of myelosuppression. 

## 3. Discussion

Any patient evaluated in the ED and found to have a high lactate raises mental alarm bells within any clinician, and this pediatric leukemic patient certainly was no exception. Elevated lactate in the ED can be an important predictor for mortality in cancer patients. Maher et al. demonstrated that, for cancer patients, there was an increased risk of mortality—reaching statistical significance in later time intervals (day 7 and day 30) [10]. This patient presented with hypoglycemia and an impressively high anion gap metabolic acidosis with elevated lactate. The initial concern was that these laboratory numbers indicated hypoxia and subsequent tissue damage. 

Although there is no strict cutoff for lactate levels, generally a lactate >4 mmol/L is classified as elevated [10]. Lactate may be elevated for two broad reasons: (1) elevated production under times of metabolic stress or (2) reduced excretion [10]. The first reason for elevated lactate is most often due to tissue necrosis, either regional or global, which has many etiologies [10]. This often includes sepsis and hemorrhagic shock, among other causes. The second reason for elevated lactate—reduced excretion—would be an indicator of compromised liver and kidney function [10]. Lactate is primarily hepatically cleared with only minor assistance from the kidneys. The liver metabolizes approximately 70% to 75% of circulating lactate, while the kidney accounts for 20–30% [10]. Therefore, patients with liver dysfunction may have an elevated lactate due to insufficient clearance. 

For this patient, reduced hepatic clearance was found to best explain his laboratory findings. The metabolic acidosis was likely secondary to an elevated lactate, and the elevated lactate was secondary to impaired clearance due to liver dysfunction. The etiology of his liver dysfunction is best explained by the hepatotoxicity of 6MP, a known adverse effect of the medication. 

Further evaluation did not reveal a source of the elevated production of lactate; therefore, the prevailing concern was toxicity from mercaptopurine, supported by his remarkably elevated 6MMPNs [6]. He appeared well and in no acute distress, a marked difference from patients with similar degrees of metabolic acidosis. The patient’s labs—severe hypoglycemia, ketoacidosis, and elevated lactate—indicated severe disease, yet these values contradicted the physical exam, which only consisted of dry mucous membranes, tachycardia, and mild abdominal discomfort. The non-focal physical exam combined with an unrevealing diagnostic work-up is unusual in a patient with such impressive laboratory values, regardless of the etiology. Additionally, the degree of hypoglycemia is unusual in an older child, particularly as older children have a more developed glucose storage capacity [11,12]. This case, therefore, may add nuance to the existing literature, as the patient was not severely ill on presentation and was of an older age, despite his severe hypoglycemia and acidosis. 

Patients who experience hypoglycemia while taking 6MP are often those who administer the medicine in the evening [4,6,11]. Since nausea is a common side effect of 6MP, it is thought that nighttime administration mitigates this unpleasant side effect for these patients [4]. However, it is known that nighttime fasting increases the risk of hypoglycemia via blockade of the liver converting alanine to pyruvate by the metabolite 6MMP [4]. This patient confirmed that he was taking 6MP at night, and it is likely that his evening administration of 6MP contributed to his hypoglycemic presentation. Considering alternative etiologies, many children undergoing chemotherapy may lose weight, which may decrease carnitine levels or increase the risk of developing ketotic hypoglycemia, yet this patient had not experienced recent weight loss. Additionally, many chemotherapy protocols include steroids, such that adrenal insufficiency should also be considered in children with hypoglycemia undergoing chemotherapy. This patient had an appropriately elevated cortisol level, making adrenal insufficiency unlikely.

Typical side effects of 6MP include myelosuppression, hepatotoxicity, nausea, vomiting, poor appetite, and skin rash [6]. These common side effects are managed supportively, and 6MP should be temporarily halted if the patient experiences an unacceptable degree of myelosuppression or hepatotoxicity. Rarely, symptomatic hypoglycemia is an attributed side effect, documented in 7% of pediatric patients receiving 6MP [13]. The metabolite 6MMP is also thought to contribute to hypoglycemia in patients taking 6MP [4]. 6MMP, a metabolite of 6MP produced by the enzyme TPMT, is a known hepatotoxin [5]. In this patient, the markedly high 6MMPN level above the limit of quantification and concurrent hypoglycemia provided further evidence that the elevated 6MMPN level was the etiology for hypoglycemia [5]. His 6MMPNs levels were trended for one year while on allopurinol and were not significantly elevated (see Table 2). During this year, he did not experience another hypoglycemic episode after the addition of allopurinol, which efficiently reverted his metabolic profile to favor the production of 6TGNs over 6MMPNs.

**Figure 1:**
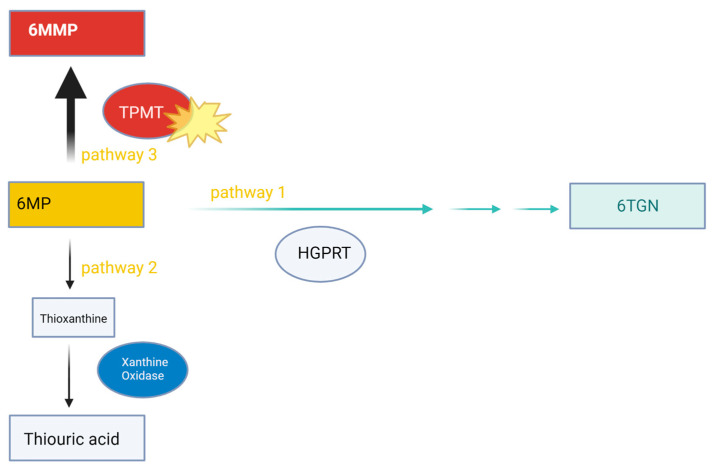
6-MP Metabolism in this patient. Three possible pathways for 6-MP [14]: 1. Hypoxanthine phosphoribosyltransferase (HGPRT) metabolizes it to result in 6TGN (therapeutic effect); 2. metabolized by xanthine oxidase to result in thiouric acid excreted in urine; 3. metabolized by TPMT to make 6MMP, a hepatotoxic substance. Increased activity of TPMT favors the production of 6MMP. Rapid 6MP metabolizer profile favors TPMT pathway. Nighttime administration of 6MP also favors TMPT activity, although this mechanism is not well understood.

**Figure 2:**
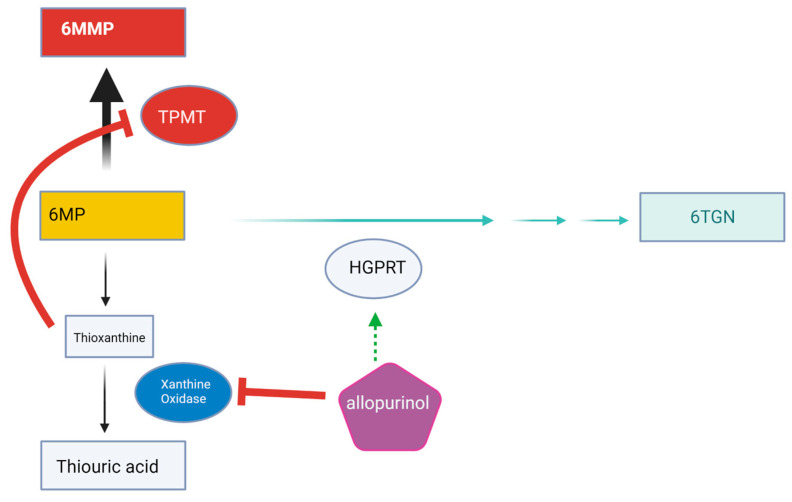
6-MP Metabolism with the addition of allopurinol [14,15]: Allopurinol inhibits xanthine oxidase, leading to a buildup of thioxanthine, which inhibits TPMT. Allopurinol also upregulates HGPRT, which favors production of anti-leukemic 6TGNs [6]. 6MP-associated hypoglycemia with metabolic acidosis is a rare side effect, but it may be more prevalent than the literature suggests [15]. Symptoms of nausea, vomiting, and somnolence mirror typical side effects of many chemotherapies. Perhaps these typical chemotherapy symptoms are actually evidence of subclinical hypoglycemia, which would indicate that this phenomenon is more common than currently recognized. Moreover, Shih et al. reported that patients with irritable bowel syndrome receiving 6MP with preferential 6MMP metabolism experienced flu-like symptoms of nausea, vomiting, and fatigue [16]. This may be further evidence of undiagnosed symptomatic hypoglycemia.

It is thought that the hepatotoxic effects of 6MMP, combined with metabolic changes from cancer, may increase the risk of hypoglycemia in leukemic patients receiving 6MP [13]. TPMT genetic polymorphisms may yield increased activity, leading to an increased production of 6MMP and therefore increased hypoglycemia risk [13] (see Figure 1). Additionally, TPMT activity is induced by certain drugs, including chemotherapy such as 6MP [14]. As seen with this patient’s case, allopurinol has been reported to reverse mercaptopurine-induced hypoglycemia in children with ALL by modulating 6MP metabolism [15] (see Figure 2).

## 4. Conclusions

This case highlights the importance of understanding the pathophysiology of hypoglycemia with high anion gap metabolic acidosis in an otherwise well-appearing child. Hypoglycemia is a serious side effect of 6MP, and, as this case demonstrates, a patient’s presentation can be variable in the pediatric population. Presentation can range from serious complications such as seizures to more indolent symptoms such as fatigue or nausea. In this case, although the elevated lactate, low pH, and ketoacidosis would suggest a more ill-appearing child, the patient was completely well appearing. He was discharged after evidence of normalized chemistries. As it was felt that the markedly elevated 6MMPNs were the cause for his hypoglycemic episode, given his lack of other underlying risk factors, 6MP was reinitiated at a lower dose along with allopurinol, which efficiently reverted his metabolic profile to favor the production of 6TGNs over 6MMPNs. Additional measures to decrease the risk of hypoglycemia include a morning administration of 6MP and frequent snacks. His case emphasizes that a high index of suspicion is needed to ensure the adequate detection and prompt treatment of this side effect given the variable presentation.

## Figures and Tables

**Table 1:** Laboratory studies on presentation.

Venous blood gas	pH 7.07 (nl 7.32–7.43), CO_2_ 33 mmHg (nl 40–60 mmHg), bicarbonate 9 mmol/L (nl 22–27 mmol/L)
Platelet count	50 × 10^9^/L (nl 150–450 × 10^9^/L)
Absolute neutrophil count	320 × 10^9^/L (nl 2500–6000 × 10^9^/L)
Glucose	30 mg/dL (nl 70–100 mg/dL)
Lactate	8.7 mmol/L (nl < 2.0 mmol/L)
Beta hydroxybutyrate	2.8 mmol/L (nl 0–0.5 mmol/L)
Aspartate aminotransaminase	197 IU/L (nl 10–40 IU/L)
Alanine aminotransferase	256 IU/L (nl 10–40 IU/L)
Total Bilirubin	1.1 mg/dL (nl < 1.0 mgd/dL)
6TGNs	585 p/mol/8 × 10^8^ RBC (nl 235–450 p/mol/8 × 10^8^ RBC)
6MMPNs	>52,320 pmol/8 × 10^8^ RBC (nl ≤ 5700 pmol/8 × 10^8^ RBC)

**Table 2:** 6-MMP (nl < 5700 pmol/8 × 10^8^ RBC) level trend in the patient from November 2021–November 2022.

Date	6-MMP Level
3 November 2021	>52,320
3 January 2022	1831
24 February 2022	<422
4 November 2022	8768

## Data Availability

Data are contained within the article.
